# Interval Cancer in Population-Based Colorectal Screening Programmes: Incidence and Characteristics of Tumours

**DOI:** 10.3390/cancers16040769

**Published:** 2024-02-13

**Authors:** Mercedes Vanaclocha-Espí, Marina Pinto-Carbó, Josefa Ibáñez, María José Valverde-Roig, Isabel Portillo, Francisco Pérez-Riquelme, Mariola de la Vega, Susana Castán-Cameo, Dolores Salas, Ana Molina-Barceló

**Affiliations:** 1 Foundation for the Promotion of Health and Biomedical Research in the Valencian Community (FISABIO)–Public Health, 46020 Valencia, Spainana.molina@fisabio.es (A.M.-B.); 2 General Directorate of Public Health, Valencian Community, 46020 Valencia, Spain; 3 The Basque Health Service, 48011 Bilbao, Spain; 4 General Directorate of Public Health, Murcia Region, 30008 Murcia, Spain; 5 General Directorate of Assistance Programmes, 38071 Santa Cruz de Tenerife, Spain

**Keywords:** colorectal cancer, screening, faecal immunochemical test, personalisation strategies, decision aids

## Abstract

**Simple Summary:**

Faecal occult blood test interval cancer is one of the potential harms of colorectal cancer screening programmes. These tumours are diagnosed in people who have previously received a negative result in a screening test. The objective of this study was to evaluate interval cancer rates in colorectal cancer screening programmes in Spain, assess their risk factors and compare the characteristics of screen-detected and interval cancer tumours. Identifying interval cancer rates as well as the associated factors would make it possible to improve screening strategies and identify personal variables related to the occurrence of interval cancer. Furthermore, studying the characteristics that distinguish interval cancers from screen-detected cancers would help us understand the nature of these tumours. These results would help evaluate and improve colorectal cancer screening programmes, increase their quality and minimise interval cancer in such programmes.

**Abstract:**

The objective of this study is to evaluate interval cancer (IC) in colorectal cancer (CRC) screening, which is CRC diagnosed in an individual after having received a negative faecal occult blood test and before the next invitation to participate in screening. A follow-up study was conducted on a cohort of participants in the first three screening rounds of four colorectal cancer screening programmes in Spain, *n* = 664,993. A total of 321 ICs and 2120 screen-detected cancers (SCs) were found. The IC and SC rates were calculated for each guaiac (gFOBT) or immunochemical (FIT) test. A Cox regression model was used to estimate the hazard ratios (HR) of IC risk factors. A nested case–control study was carried out to compare IC and SC tumour characteristics. The IC rate was 1.16‰ with the gFOBT and 0.35‰ with the FIT. Men and people aged 60–69 showed an increased probability of IC (HR = 1.81 and HR = 1.95, respectively). There was a decreased probability of IC in individuals who regularly participated in screening, HR = 0.62 (0.47–0.82). IC risk gradually rose as the amount of Hb detected in the FIT increased. IC tumours were in more advanced stages and of a larger size than SC tumours, and they were mostly located in the cecum. These results may play a key role in future strategies for screening programmes, reducing IC incidence.

## 1. Introduction

Colorectal cancer (CRC) is the second most common cancer in women and the third most common in men, and it is also the second most common cause of cancer deaths behind lung cancer in Spain [1]. CRC screening programmes (CRCSP) aimed at the medium-risk population (women and men aged between 50 and 74) are one of the main strategies to reduce the mortality and incidence of this type of tumour and have the purpose of detecting early-stage tumours and CRC precursor lesions (adenomas) [2,3,4]. A faecal occult blood test (FOBT) carried out every two years, followed by a colonoscopy to confirm the diagnosis, is one of the recommended strategies [5]. To evaluate the impact of these programmes in terms of reducing mortality and incidence, they must have been in place for a long, uninterrupted period of time [5]. Certain short- and medium-term indicators of the benefits and harms have been defined to help predict the long-term impact of these programmes [5].

One of the potential harms of CRCSPs that periodically use the FOBT for screening is FOBT interval cancer (IC) [5]. IC is cancer of the colon or rectum that is clinically diagnosed in an individual after having received a negative FOBT screening result and before being invited to participate in the programme again [5]. Failure to detect the cancer could be due to various causes, for example, if the tumour bleeds intermittently, if the screening test fails, or if the tumour does not exist at the time of screening. For this reason, it is important to analyse the anatomical and pathological features of these types of cancers.

To identify IC, CRCSP and cancer registries must be carefully linked to detect cancers that are diagnosed in CRCSP participants who received a negative FOBT result. Few studies assess the IC rate, even though it is a key indicator of CRCSP quality. The IC rate is directly linked to FOBT sensitivity, and the sensitivity of different types of tests for detecting CRC or adenomas varies. Several studies show that the faecal immunochemical test (FIT) is more sensitive than the guaiac FOBT (gFOBT) [6,7,8].

As expected, due to the variations in FOBT sensitivity, differences can be seen in the IC rate depending on the type of FOBT used [9,10]. Wieten E et al.’s study compared IC incidence following a negative gFOBT or FIT result, and the rates were 34 and 20 per 100,000 individuals, respectively [9].

The FIT quantifies the amount of Hb in faeces. When this type of FOBT is used in screening, a cut-off point is applied to determine whether a result is positive or negative. It has also been demonstrated that the established cut-off point affects the IC rate, as can be seen in a previous study that observed how different FIT cut-off points lead to changes in the IC rate [11]. Other variables such as sex and age have been linked to the occurrence of IC, with studies showing that IC is more frequent in men and older populations [12,13].

In contrast to screen-detected CRC (SC), IC diagnoses are mostly based on symptoms, and tumours are therefore likely to have a worse prognosis than those detected in screening. Some studies compare the characteristics of IC and SC tumours and observe differences in tumour location and stage [12,13,14,15,16]. Some studies observe a higher percentage of IC than SC in the right side of the colon [12,14,16], while others observe a higher percentage of IC in the rectum [13,14,15,16]. As regards tumour stage, ICs have been associated with more advanced stages of cancer [12,13,14,15,16].

Since determining that IC is a key aspect when evaluating CRCSP, one of the aims of this study is to estimate IC rates in the CRCSP implemented in Spain. Furthermore, this study aims to compare the anatomical and pathological characteristics of IC and SC tumours and increase knowledge on IC risk factors.

## 2. Materials and Methods

### 2.1. CRIBEA-CIN Project

CRIBEA-CIN is a multicentre cohort study aimed at assessing short- and medium-term indicators of the benefits and harms of CRCSP. For this purpose, a cohort of participants in four CRCSPs implemented in Spain (in the Canary Islands, the Basque Country, the Region of Murcia and the Community of Valencia) between 2006 and 2012 was monitored.

The CRCSPs involved in this study perform an FOBT (gFOBT or FIT) every two years. The cut-off point for the FIT was 20 µg/g and a colonoscopy was offered as a confirmatory diagnostic test. The characteristics of these programmes and the outcomes associated with this cohort can be found in previous studies by Vanaclocha-Espí et al., 2021 and 2019 [11,17].

This study analysed 664,693 gFOBT or FIT screening tests. Cases of IC within the two years following participation were identified in this cohort, in accordance with the recommendations of the European Guidelines [5] and the National Health Service [18]. The cancer registries of each participating region were used to identify IC, and the cases were confirmed by performing an active search in the corresponding regional hospital information records. By definition, ICs are cancers that are diagnosed in an individual outside of screenings, after having received a negative FOBT result and before screening is next due [5]. In this study, ICs diagnosed after 24 months were excluded for the analysis (*n* = 39).

**Scheme 1:**
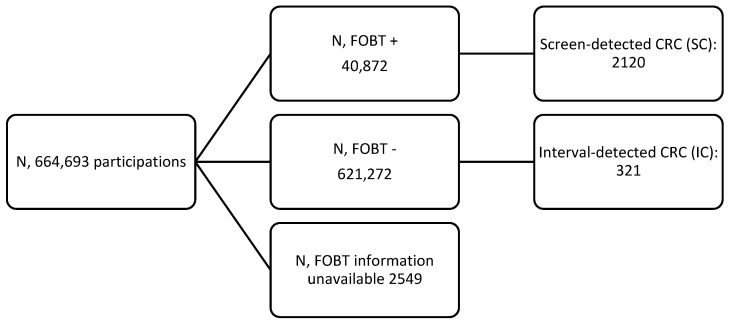
Study population diagram. Acronyms: faecal occult blood test (FOBT), colorectal cancer (CRC).

In this cohort, 2120 SCs and 321 ICs were detected (Scheme 1). A nested case–control study was performed to compare ICs and SCs. The ICs were the cases and the SCs were the controls. A search for information on tumour characteristics was carried out for all cases and controls.

### 2.2. Study Variables

Personal characteristics: sex (male/female); age at the time of the FOBT (50–59/60–69). Organisational characteristics of the CRCSP: type of FOBT (gFOBT/FIT); type of participation (initial: population that has not previously participated in the programme/subsequent: population that has previously participated in the programme); amount of occult haemoglobin in faeces in the test immediately prior to IC detection (Hb/g) (this variable was categorised into 5 levels: <1 µg/g was considered an undetectable amount, and the variable was categorised in quartiles between 1 µg/g and 20 µg/g). Tumour characteristics: location (cecum/ascending/transverse/descending/sigmoid or rectum); tumour size in mm; morphology (adenocarcinoma NOS (not otherwise specified)/other); and tumour stage (I/II/III/IV).

### 2.3. Statistical Analysis

The study sample was described and the SC and IC rates per 1000 FOBTs were calculated. The rates were obtained for each type of FOBT and participation. Kaplan–Meier survival curves were used to estimate the cumulative probability of IC occurring in individuals who had received a negative FOBT result, and the distribution of these curves was compared for the variables sex, age and amount of Hb in faeces (only FIT cases) using a logrank test. A Cox regression model was used to study the association of personal characteristics and screening history with IC, and the time scale was the period from when the FOBT was carried out to IC diagnosis. The results are shown as hazard ratios (HR). The models were calculated for all negative FOBTs (gFOBT and FIT) and for FITs only. Logistic regression was used to compare the personal characteristics and anatomical and pathological tumour characteristics of SC and IC cases. These results are shown as odds ratios (OR) and 95‰ confidence intervals (CI). Statistical analyses were performed with the R statistical programme, and a significance level of 0.05 was considered when interpreting the results.

## 3. Results

The IC rate was 1.16 per 1000 gFOBTs performed and 0.35 per 1000 FITs performed. A total of 63.9‰ of ICs were diagnosed between 12 and 24 months after taking the FOBT. A total of 33.9‰ of ICs diagnosed following a FIT showed an undetectable quantity of Hb in faeces (<1 μg/g) in the screening. As regards participation type, the IC rates were 1.24 per 1000 gFOBTs and 0.39 per 1000 FITs in the initial screening, and 0.93 per 1000 gFOBTs and 0.25 per 1000 FITs in the subsequent screening (Table 1).

**Figure 1:**
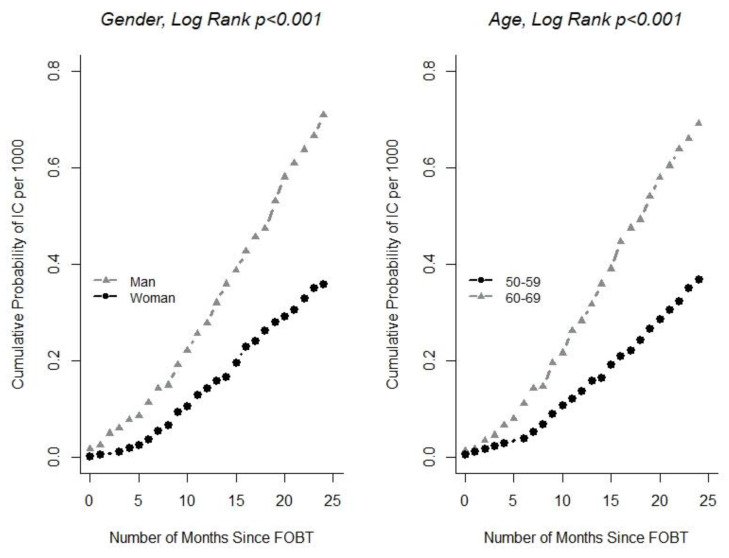
Cumulative risk of interval cancer (IC) by sex and age.

**Figure 2:**
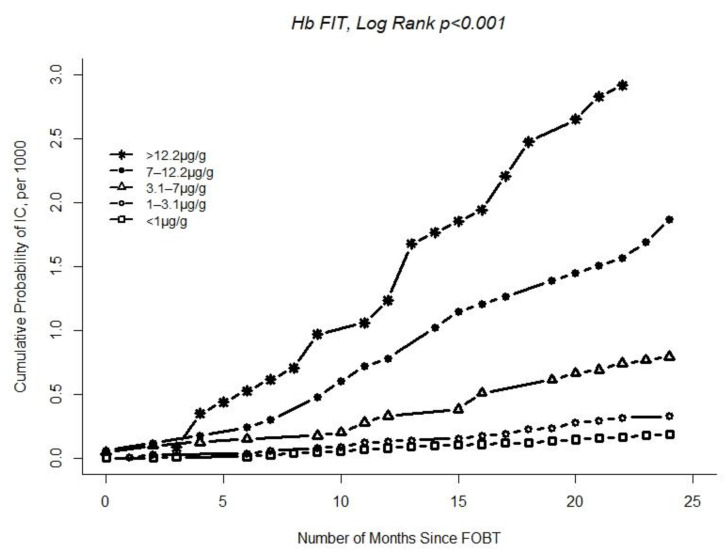
Cumulative risk of IC according to the amount of blood detected in faeces in the CRCSP FIT.

Figure 1 shows the cumulative probability of developing IC by sex and age. The survival distribution showed statistically significant differences in the two variables (logrank *p*_value < 0.001). Men had a higher cumulative probability of developing IC than women (0.7 vs. 0.4‰ at 24 months), and individuals aged between 60 and 69 had a higher cumulative probability than individuals aged between 50 and 59 (0.69 vs. 0.37‰ at 24 months). Figure 2 shows an increase in the cumulative probability of IC when the amount of Hb in faeces is close to 20 μg/g (0.1‰ for 0 μg/g, 0.3‰ for 1–3.1 μg/g, 0.8‰ 3.1–7 μg/g, 2‰ for 7–12.2 μg/g and 3‰ for >12.2 μg/g). These differences were statistically significant (logrank *p*_value < 0.001).

The HR of the survival analyses for IC are shown in Table 2. The older age group and males showed a higher risk of IC (HR = 1.81 (1.44–2.27) and HR = 1.95 (1.56–2.45), respectively). The risk was lower when the FIT was used (HR = 0.34 (0.27–0.43)). The amount of Hb detected in faeces by the FIT showed an increased risk of developing IC, compared to <1 μg/g as the reference measure HR = 1.75 (1.15–2.68) for 1–3.1 μg/g, HR = 3.91 (2.55–6.00) 3.1–7 μg/g, HR = 9.03 (5.88–13.86) 7–12.2 μg/g and HR = 13.73 (9.01–20.91) for>12.2 μg/g. 

Table 3 shows the results of the comparison between the IC and control (SC) groups. Compared to SC, IC was more frequently found in the caecum vs. the sigmoid or rectum, OR = 2.92 (1.62–5.12). Additionally, IC was more frequently found in more advanced stages of the disease (larger size and stage IV), and there were no statistically significant differences according to sex, age and type of participation. There were no statistically significant differences in tumour location for IC diagnosed during the first 12 months after participation in screening. In addition, a higher risk of morphologies other than adenocarcinoma NOS was observed in this group, OR = 3.65 (1.35–8.81). For IC diagnosed between 12 and 24 months after screening, significant differences in location were observed, and IC was more frequently found in the caecum, OR = 3.49 (1.34–4.76).

## 4. Discussion

This study provides information on IC and SC rates in population-based screening programmes for each type of FOBT (FIT and gFOBT). It shows that the likelihood of developing IC increases from the time that the last screening test is taken to when the next invitation to participate is received 24 months later, with differences according to sex, age and the amount of Hb detected in faeces. Furthermore, we observed anatomical and pathological differences between IC and SC.

The IC rate differed depending on the type of FOBT used. Namely, the FIT was associated with a lower IC rate compared to the gFOBT, which indicates that the former has higher sensitivity. In addition, we observed a higher SC rate when this test is used. These results are in line with existing scientific literature on both tests’ ability to detect CRC, which shows that the FIT offers higher positive predictive value for CRC detection and higher sensitivity than the gFOBT [9,19,20,21,22]. With the FIT, the SC rate in subsequent screening was lower, in line with another study that showed a lower CRC risk in a subsequent round of screening using the FIT [23]. The differences in IC rates for the different types of FOBT seem to indicate that IC sometimes occurs as the screening test fails to detect the cancer or lesion, and IC cases could therefore be minimised in part if the more sensitive FOBT were used.

We observed differences in the likelihood of developing IC based on the amount of Hb found in faeces in the last screening test before IC is detected. This reinforces the conclusions of other studies that screening programmes could be further optimized by stratifying risk, taking into account the amount of Hb detected in faeces in previous rounds of screening [24]. Differences in the amount of Hb in faeces suggest that changes in the cut-off point for determining a positive result could influence the IC rate, as observed in other research [11,25]. The likelihood of IC is substantially lower when undetectable Hb concentrations of 0 μg/g are found in screening tests. Nonetheless, an undetectable amount of Hb was found in the case of almost 24% of ICs diagnosed following screening with the FIT, although it is unknown whether these ICs were present at the time of screening.

With respect to Hb in faeces in previous rounds of screening, one study observed that CRC is less likely to be detected in a subsequent round of screening and that individuals with an undetectable amount of Hb in faeces (0) in the previous round of screening presented a lower risk than individuals with a detectable amount of Hb in faeces (>0) [24]. In our study, we observed that screening results influence the risk of developing IC, thereby supporting the idea of this study.

Our study shows that men and older people have a higher risk of IC, in line with scientific evidence concluding that both sex and age are CRC risk factors [22]. Our results suggest that this relationship is also true for cancers not detected by FOBT screening.

Other studies on CRCSP have shown that IC is found more frequently than SC on the right side of the colon with both the gFOBT and FIT [13,16,26]. Specifically, one study analysed the sensitivity of the FIT as related to tumour location and found lower sensitivity when the tumour is located on the right side of the colon [16]. Our results are consistent with these findings, as IC was found more frequently in the caecum, the farthest part of the colon, which suggests that FOBT sensitivity may vary depending on tumour location.

Other studies have revealed that IC tumours are in more advanced stages and of a larger size than SC tumours [9,13,27,28,29]. Our results in this regard are consistent with their findings and conclude that the characteristics associated with IC suggest a worse prognosis.

Finally, as expected in this study, the most frequent morphology in CRC (SC and IC) is adenocarcinoma NOS (90%). Furthermore, the results of our study show clear differences in the morphology of SC and IC tumours. Specifically, IC cases (especially those that appear during the first year after participation in screening) more frequently have morphologies other than adenocarcinoma NOS. These findings are in line with the study of Blanks et al., whose results suggest that rarer morphologies such as mucinous and signet ring are more likely to be detected as IC [30]. A comparison of IC vs. SC showed no differences according to sex and age, in line with other studies [28,30,31].

## 5. Conclusions

This research provides a comprehensive categorisation of IC and SC. On the one hand, we have categorised ICs as tumours with a worse prognosis and more specific morphologies, suggesting that these tumours may be associated with more aggressive behaviour. On the other hand, we have observed differences in the IC rate depending on the type of FOBT used, which suggests that some ICs may not be diagnosed in screening due to limitations in FOBT sensitivity. Furthermore, we have observed differences in the risk of developing IC according to sex, age and the amount of Hb detected in faeces with the FIT, which provides relevant information that can be used to personalise screening and help minimise IC incidence.

## Figures and Tables

**Table 1:** Number of screen-detected cancers and interval cancers with rates per 1000 FOBTs performed.

	Whole Sample		gFOBT		FIT	
	N	Rate *	N	Rate *	N	Rate *
People screened	488,136					
FOBTs performed	664,693		106,064		558,629	
In initial screening	488,136		79,137		408,999	
In subsequent screening	176,557		26,927		149,630	
SC	2120	3.19	144	1.36	1976	3.54
In initial screening	1704	3.49	106	1.34	1598	3.91
In subsequent screening	416	2.36	38	1.41	378	2.53
IC	321	0.48	123	1.16	198	0.35
In initial screening	258	0.52	98	1.24	160	0.39
In subsequent screening	63	0.36	25	0.93	38	0.25
Time to diagnosis (med ± SD)	14.04 ± 6.38		14.43 ± 6.37		13.80 ± 6.38	
<12 months	116 (36.1)		41 (33.3)		75 (37.9)	
12–24 months	205 (63.9)		82 (66.7)		123 (62.1)	
IC according to Hb in negative FIT (6 unknown)						
<1 μg/g					65 (33.9%)	
1–3.1 μg/g					32 (16.7%)	
3.1–7 μg/g					31 (16.1%)	
7–12.2 μg/g					31 (16.1%)	
>12.2 μg/g					33 (17.2%)	

* Rate per 1000 tests. Acronyms: guaiac faecal occult blood test (gFOBT), immunochemical faecal occult blood test (FIT), screen-detected colorectal cancer (SC), interval cancer (IC), standard deviation (SD).

**Table 2:** Cox model: risk of IC in screening with a negative FOBT result.

Negative FOBTs				Only Negative FITs		
	No. Negative FOBT	IC	HR (95% CI)	No. Negative FIT	IC	HR (95% CI)
Age						
50–59	334,293	123	1	288,262	83	1
60–69	286,619	198	1.81 (1.44–2.27)	229,843	115	1.68 (1.26–2.24)
Sex						
Female	339,859	122	1	283,948	74	1
Male	281,053	199	1.95 (1.56–2.45)	234,157	124	1.84 (1.37–2.46)
FOBT type						
Guaiac	102,807	123	1			
FIT	518,105	198	0.34 (0.27–0.43)			
Type of participation						
Initial	454,021	258	1	377,547	160	1
Subsequent	166,864	63	0.62 (0.47–0.82)	140,531	38	0.65 (0.45–0.92)
Hb in faeces						
<1 μg/g				341,837	65	1
1–3.1 μg/g				97,043	32	1.75 (1.15–2.68)
3.1–7 μg/g				38,950	31	3.91 (2.55–6.00)
7–12.2 μg/g				16,567	31	9.03 (5.88–13.86)
>12.2 μg/g				11,306	33	13.73 (9.01–20.91)

Multivariate time-dependent Cox models. The HRs for each variable were adjusted for all other variables in the table. Negative FOBTs include gFOBTs and FITs. Acronyms: guaiac faecal occult blood test (gFOBT), immunochemical faecal occult blood test (FIT), interval cancer (IC), hard ratio (HR), confidence interval (CI).

**Table 3:** Characteristics of IC vs. SC.

	IC vs. SC			IC < 12 m vs. SC		IC 12–24 m vs. SC	
	IC *n* (%)	SC *n* (%)	OR (95% CI)	IC *n* (%)	OR (95% CI)	IC *n* (%)	OR (95% CI)
Sex							
Female	120 (37.4)	715 (33.7)	ref	49 (38.6)	ref	71 (36.6)	ref
Male	201 (62.6)	1405 (66.3)	0.98 (0.65–1.49)	78 (61.4)	1.14 (0.62– 2.17)	123 (63.4)	0.92 (0.56–1.55)
Age							
50–59	123 (38.3)	790 (38.3)	Ref	46 (36.2)	Ref	77 (39.7)	ref
60–69	198 (61.7)	1330 (61.7)	0.85 (0.57–1.27)	81 (63.8)	1.00 (0.54–1.86)	117 (60.3)	0.80 (0.5–1.31)
Type of screening							
Initial	258 (80.4)	1704 (80.4)	ref	100 (78.7)	ref	158 (81.4)	Ref
Subsequent	63 (19.6)	416 (19.6)	1.36 (0.79–2.26)	27 (21.3)	2.02 (0.96–4.04)	36 (18.6)	0.96 (0.45–1.89)
Location							
Sigmoid or rectum	177 (60.0)	1095 (69.0)	ref	66 (56.9)	ref	111 (62.0)	ref
Caecum	49 (16.6)	92 (5,8)	2.92(1.62–5.12)	16 (13.8)	1.82 (0.62–4.60)	33 (18.4)	3.49 (1.34–4.76)
Ascending	27 (9.2)	126 (7.9)	1.05 (0.49–2.06)	15 (12.9)	1.96 (0.79–4.47)	12 (6.7)	0.42 (0.10–1.25)
Transverse	30 (10.2)	179 (11.3)	1.07 (0.55–1.94)	13 (11.2)	1.33 (0.51–3.09)	17 (9.5)	0.96 (0.40–2.03)
Descending	12 (4.1)	97 (6.0)	0.33 (0.08–1.09)	6 (5.2)	0.48 (0.07–2.04)	6 (3.4)	0.31 (0.05–1.27)
Tumour size, mm			1.20 (1.16–1.25)		1.18 (1.14–1.24)		1.19 (1.15–1.25)
Morphology							
Adenocarcinoma NOS	269 (93.4)	2003 (97.8)	ref	107 (92.2)	ref	162 (94.2)	ref
Other *	19 (6.6)	45 (2.2)	2.37 (1.11–4.79)	9 (7.8)	3.65 (1.35–8.81)	10 (5.8)	1.62 (0.55–4.06)
Stage							
I	47 (18.1)	998 (50.9)	ref	16 (14.5)	ref	31 (20.7)	ref
II	58 (22.3)	382 (19.5)	2.08 (1.17–3.73)	30 (27.3)	3.03 (1.29–7.60)	28 (18.7)	1.40 (0.66–2.94)
III	81 (31.2)	450 (22.9)	2.51 (1.48–4.35)	30 (27.3)	2.14 (0.89–5.43)	51 (34.0)	2.49 (1.34–4.76)
IV	74 (28.5)	131 (6.7)	4.55 (2.39–8.62)	34 (30.9)	5.56 (2.06–15.27)	40 (26.7)	3.90 (1.81–8.30)

Multivariate models. The OR for each variable was adjusted for all other variables in the table. * medullary carcinoma NOS, serrated adenocarcinoma, signet ring cell carcinoma, mucinous carcinoma, undifferentiated carcinoma, cribriform adenocarcinoma. Acronyms: interval cancer (IC), screen-detected colorectal cancer (SC), odds ratio (OR), confidence interval (CI).

## Data Availability

The datasets presented in this article are not readily available due to legal restrictions on sharing the data set, as regulated by the Valencia regional government by means of legal resolution by the Valencia Health Agency (2009/13312), which forbids the dissemination of data to third parties. Available from: https://www.san.gva.es/ca/web/investigacio/programas-normativa-y-legislacion (accessed on 12 February 2024). Upon request, authors can obtain access to the databases to verify the accuracy of the analysis or the reproducibility of the study. Requests to access the data sets should be directed to the Management Office of the Data Commission of the Valencia Health Agency. Available from: https://www.san.gva.es/ca/web/investigacio/acceso-a-la-aplicación (accessed on 12 February 2024).
